# Effectiveness of a Blended Web-Based Intervention on Return to Work for Sick-Listed Employees With Common Mental Disorders: Results of a Cluster Randomized Controlled Trial

**DOI:** 10.2196/jmir.4097

**Published:** 2015-05-13

**Authors:** Daniëlle Volker, Moniek C Zijlstra-Vlasveld, Johannes R Anema, Aartjan TF Beekman, Evelien PM Brouwers, Wilco HM Emons, A Gijsbert C van Lomwel, Christina M van der Feltz-Cornelis

**Affiliations:** ^1^Trimbos InstituteNetherlands institute of mental health and addictionUtrechtNetherlands; ^2^Tilburg UniversityTranzo DepartmentTilburgNetherlands; ^3^VU University Medical CenterDepartment of Public and Occupational Health and the EMGO Institute for health and care research (EMGO+)AmsterdamNetherlands; ^4^VU University Medical CenterDepartment of Psychiatry and the EMGO+ Institute for Health and Care ResearchAmsterdamNetherlands; ^5^Tilburg UniversityDepartment of Methodology and StatisticsTilburgNetherlands; ^6^Achmea Disability InsuranceTilburgNetherlands; ^7^GGZ BreburgTopClinical Centre for Body, Mind and HealthTilburgNetherlands

**Keywords:** occupational health, randomized controlled trial, mental health, depression, anxiety, sick leave

## Abstract

**Background:**

Common mental disorders are strongly associated with long-term sickness absence, which has negative consequences for the individual employee’s quality of life and leads to substantial costs for society. It is important to focus on return to work (RTW) during treatment of sick-listed employees with common mental disorders. Factors such as self-efficacy and the intention to resume work despite having symptoms are important in the RTW process. We developed “E-health module embedded in Collaborative Occupational health care” (ECO) as a blended Web-based intervention with 2 parts: an eHealth module (Return@Work) for the employee aimed at changing cognitions of the employee regarding RTW and a decision aid via email supporting the occupational physician with advice regarding treatment and referral options based on monitoring the employee’s progress during treatment.

**Objective:**

This study evaluated the effect of a blended eHealth intervention (ECO) versus care as usual on time to RTW of sick-listed employees with common mental disorders.

**Methods:**

The study was a 2-armed cluster randomized controlled trial. Employees sick-listed between 4 and 26 weeks with common mental disorder symptoms were recruited by their occupational health service or employer. The employees were followed up to 12 months. The primary outcome measures were time to first RTW (partial or full) and time to full RTW. Secondary outcomes were response and remission of the common mental disorder symptoms (self-assessed).

**Results:**

A total of 220 employees were included: 131 participants were randomized to the ECO intervention and 89 to care as usual (CAU). The duration until first RTW differed significantly between the groups. The median duration was 77.0 (IQR 29.0-152.3) days in the CAU group and 50.0 (IQR 20.8-99.0) days in the ECO group (hazard ratio [HR] 1.390, 95% CI 1.034-1.870, *P*=.03). No significant difference was found for duration until full RTW. Treatment response of common mental disorder symptoms did not differ significantly between the groups, but at 9 months after baseline significantly more participants in the ECO group achieved remission than in the CAU group (OR 2.228, 95% CI 1.115-4.453, *P*=.02).

**Conclusions:**

The results of this study showed that in a group of sick-listed employees with common mental disorders, applying the blended eHealth ECO intervention led to faster first RTW and more remission of common mental disorder symptoms than CAU.

**Trial Registration:**

Netherlands Trial Register NTR2108; http://www.trialregister.nl/trialreg/admin/rctview.asp?TC=2108. (Archived by WebCite at http://www.webcitation.org/6YBSnNx3P).

## Introduction

### Common Mental Disorders and Sickness Absence

Common mental disorders, such as depression, anxiety, and somatization disorders, are strongly associated with long-term sickness absence [1,2] and have negative consequences for the quality of life of the sick-listed employee. Prolonged sickness absence can lead to social isolation, income reduction, reduction of meaningful activity, and anxiety to return to work (RTW) [3,4]. The longer the duration of sickness absence, the more difficult RTW may become. In addition to the consequences of sickness absence for the individual employee, sickness absence also leads to substantial costs for society. Sickness absence due to common mental disorders leads to one-third of all disability benefits in The Netherlands [5]. Estimated annual costs of sickness absence in The Netherlands due to mental disorders are €2.7 billion [2].

### Return to Work

Several studies have shown that a reduction of common mental disorder symptoms was not enough to reduce sickness absence [6,7]. Moreover, interventions focusing on symptoms alone did not have an effect on sickness absence [6-9]. Therefore, it is important to also focus on RTW during treatment of sick-listed employees with common mental disorders. However, in Dutch social security legislation, treatment sickness certification is separated with occupational physicians playing an important role in the guidance of sickness absence while the curative sector provides treatment. Although this legislation was introduced to protect the employee, RTW is hampered as a result due to a lack of collaboration and communication between occupational physicians and the curative sector. Also, RTW is often not addressed in the treatment of sick-listed employees [10,11]. Another study showed that the occupational physicians often neither monitor symptoms nor evaluate the initiated treatment [12]. To overcome these barriers, Van der Feltz et al [13] studied a form of collaboration in which occupational physicians worked together with consultant psychiatrists in the guidance of employees with common mental disorders. Although this form of collaboration did not reveal a statistically significant reduction in the duration of sickness absence until RTW, the results were promising [13]. Vlasveld et al [14] studied the effectiveness of an even more elaborated form of collaboration, namely a collaborative care model. In this model, an occupational physician trained in this model provided the treatment for major depressive disorder and the regular occupational physician provided the guidance in sickness absence. Despite the dual focus on RTW and symptoms, the results of this study showed an improvement of depressive symptoms but not of RTW [14]. These results may reflect implementation problems, which in turn could be explained by the fact that the employees and the occupational physicians felt uncomfortable with the occupational physician in the role of treatment provider, although the occupational physicians had received specialized training [15]. Nevertheless, the dual focus on RTW and the recovery of symptoms remains important and efforts need to be made to improve that dual focus [15]. A better model could be one in which the occupational physician is supported in the referral of the employee to adequate treatment in the curative sector by decision support based on monitoring of common mental disorder symptoms of the employee. This calls for a low-access intervention, such as eHealth, including a decision aid for the occupational physician.

### Self-Efficacy

Recent studies have shown the importance of factors such as self-efficacy and the intention to resume work despite having symptoms [16-19]. Return-to-work self-efficacy (RTW-SE) is the belief that employees have in their own ability to meet the demands required to RTW [17]. Several studies have shown that RTW-SE is a predictor of actual RTW [17,18,20]. Van Oostrom et al [16] found that a workplace intervention was effective on lasting RTW only for employees who at baseline intended to RTW while still having symptoms. The results of this study suggest that a negative intention regarding RTW while having symptoms will probably hinder the RTW process and a lack of focus on factors such as RTW-SE in treatment may lead to unnecessary sickness absence. This would have important policy implications if factors such as RTW-SE could be influenced by interventions working on these cognitions.

### Web-Based Intervention

To our knowledge, no intervention exists that specifically focuses on advancing RTW and cognitions regarding RTW for sick-listed employees with common mental disorders combined with monitoring of progress in their mental health and a decision aid for the occupational physician [21]. Because there is a need for highly available, low-threshold, low-cost interventions and more than 90% of Dutch households have Internet access, a Web-based intervention was developed [22]. The intervention was named “E-health module embedded in Collaborative Occupational health care” (ECO). The aim of ECO was to guide sick-listed employees with common mental disorders to RTW. The employee follows an eHealth module, known as Return@Work, which focuses on the employees’ cognitions regarding RTW with physical or psychological symptoms and options to resume work at least on a partial basis while symptoms are still present. Also, the recovery process of the employee is monitored in the eHealth module. An integral part of the intervention is that the occupational physician of the sick-listed employee with a common mental disorder receives automated suggestions by email for referral to adequate treatment in the curative sector from a decision aid when the monitoring of symptoms indicates a lack of progress. Progress is monitored in terms of physical and mental well-being and functioning [23].

The aim of the current study was to evaluate the effects of the ECO intervention on time to RTW and mental health outcomes. It was hypothesized that the ECO intervention would lead to a faster RTW and less common mental disorder symptoms than usual care.

## Methods

### Study Design

#### Overview

The study was a 2-armed cluster randomized controlled trial. Randomization took place at the level of occupational physician. Employees in both conditions received sickness absence guidance as usual. Employees in the intervention condition received the ECO intervention in addition. The design of this study has been extensively described in Volker et al [23]. The study protocol was approved by the Medical Ethics Committee of the University Medical Center Utrecht, The Netherlands, in February 2011.

#### Randomization of Clusters

The participants were sick-listed employees in small- to medium-sized companies visiting their occupational physician at Arbo Vitale (a large occupational health service) and sick-listed employees of GGz Breburg (a large mental health service employer) visiting their occupational physician, both in The Netherlands. Cluster randomization took place at the level of the occupational physicians to prevent contamination and thus to prevent dilution of the effect. At Arbo Vitale, occupational physicians working in the same region were clustered to reduce contamination due to occupational physicians who take over each other’s caseloads when necessary. The clusters of occupational physicians were randomized by an independent statistician using a computer algorithm for randomization. Six regions (31 occupational physicians) were allocated to the ECO group and 6 regions (29 occupational physicians) were allocated to the control group.

At GGz Breburg, only 1 occupational physician was available. For this reason, a cluster crossover design was used at first with the first 100 employees approached as the control condition and subsequently the second 100 employees approached as the intervention condition. However, at the end of the planned control condition, the occupational physician was replaced with another occupational physician, with whom the intervention condition was conducted. Therefore, this can be considered as a pseudo-randomization design in GGz Breburg.

Because the occupational physicians had to guide the intervention, they could not be blinded to the group assignment after randomization. However, they participated in only 1 experimental condition: either ECO or care as usual (CAU). The research assistants and the participants were blind to the allocation when assessing the eligibility of sick-listed employees for participating in this study. If the participant met the inclusion criteria for this study and agreed to participate, the baseline questionnaire was sent by email. After the questionnaire was filled out and informed consent was given, the participant was informed by the researchers via telephone about the allocation.

#### Sample Size

A power calculation prior to the study indicated that a sample size of 200 participants was needed to have at least .80 power to detect differences in time to RTW given a hazard ratio (HR) of 1.6 [23].

### Participants

#### Recruitment Procedure

All employees on sickness absence for any cause between 4 and 26 weeks who gave informed consent were screened for depression (Patient Health Questionnaire 9-item; PHQ-9), somatization (PHQ-15), and anxiety (Generalized Anxiety Disorder 7-item; GAD-7). Employees who were considered as screen-positive on any of the 3 screening instruments were contacted by a research assistant, who was blinded to group assignment, by telephone. The research assistants checked for inclusion and exclusion criteria and provided information about the study.

Sick-listed employees who did not meet any of the exclusion criteria received the baseline questionnaire and a second informed consent form. Employees who completed the baseline questionnaire and gave their informed consent were included in the study.

#### Inclusion Criteria

Employees (aged ≥18 years) who were on sickness absence between 4 and 26 weeks and screened positive (score ≥10) on either the depression scale of the PHQ-9 and/or the somatization scale of the PHQ-15 and/or the GAD-7 were included. These instruments have shown good psychometric properties for the screening of depression, somatization, and anxiety [24-26].

#### Exclusion Criteria

Employees were excluded for participating in this study if they had insufficient knowledge of the Dutch language, were pregnant, or were involved in legal action against their employer. Furthermore, employees without access to the Internet were excluded.

### Intervention

#### ECO

**Figure 1:**
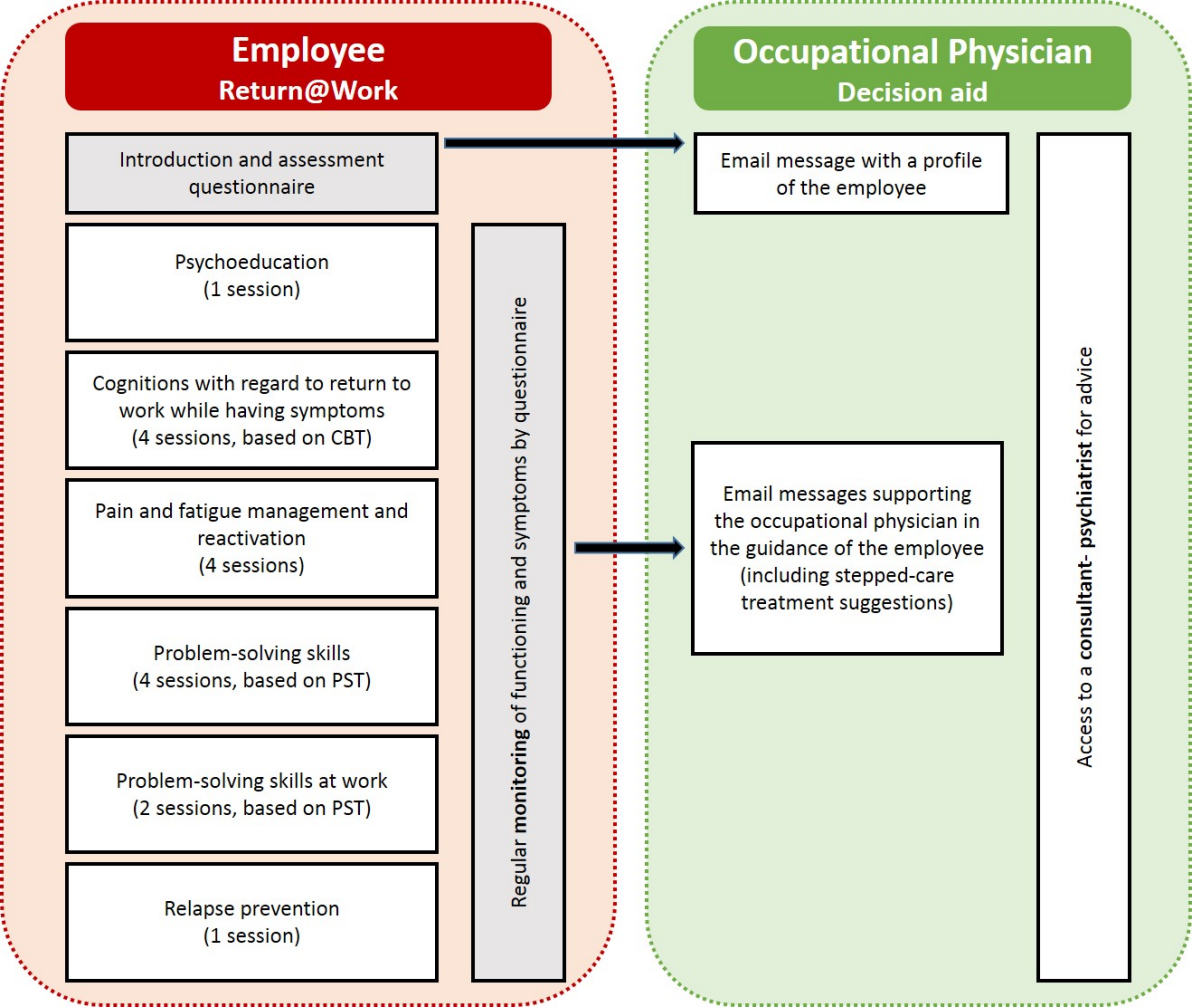
Overview of the ECO intervention.

The ECO intervention included 2 elements (an illustration of the ECO intervention can be found in Figure 1): (1) the Return@Work eHealth module and (2) an email decision aid for the occupation physician. Both are described subsequently.

#### Return@Work eHealth Module

**Figure 2:**
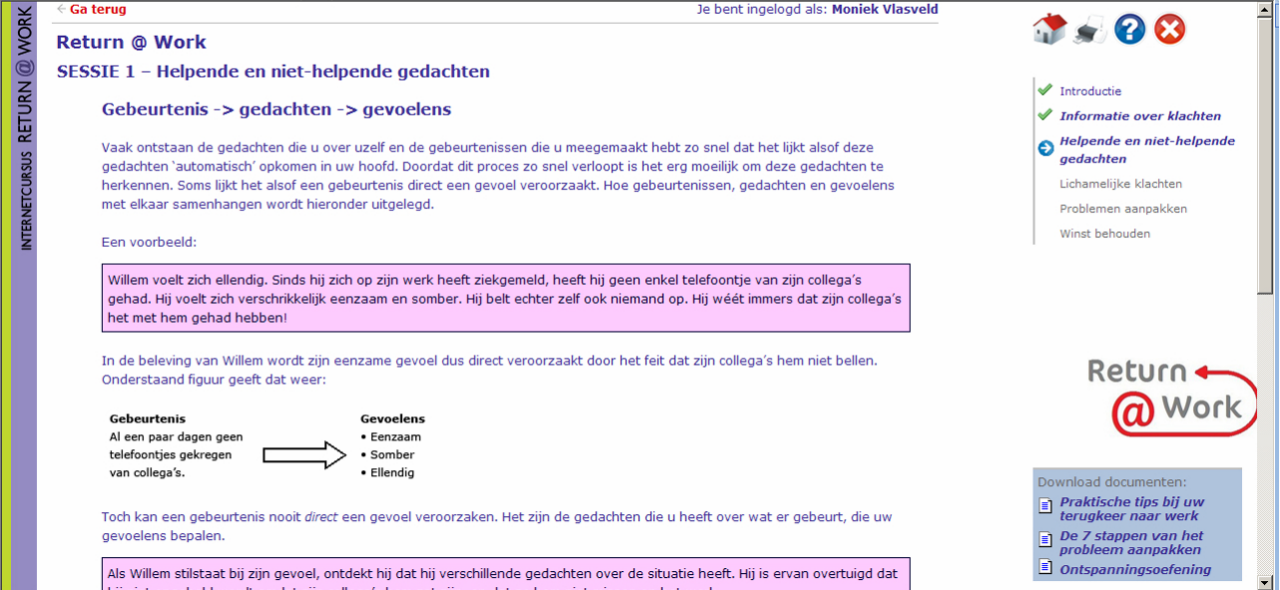
Screenshot of Return@Work.

The employee received an individual log-in code for the eHealth RTW module Return@Work. Return@Work included the following 5 modules: (1) psychoeducation, (2) a module aimed at cognitions with regard to RTW while having symptoms (based on cognitive behavioral therapy [CBT] principles), (3) a module aimed at increasing problem-solving skills with problem-solving treatment (PST) exercises, (4) a module for pain and fatigue management and for reactivation, and (5) a module for relapse prevention. In total, the modules included 16 sessions. The content of Return@Work was tailor-made to the individual employee, depending on the symptoms and cognitions about RTW of the employee. As a consequence, not every employee received all modules; therefore, the total number of sessions ranged from 6 to 17. Furthermore, functioning and symptoms were monitored on a regular basis in Return@Work. A screenshot of Return@Work can be found in Figure 2.

The employees worked through Return@Work individually, but were free to discuss topics or assignments with the occupational physician. The occupational physicians were asked to follow the guidelines of the Dutch Board for Occupational Medicine (NVAB); thus, as in usual sickness guidance, the occupational physician and employee met each other face-to-face on a regular basis [27]. The occupational physicians were instructed to inquire about the employee’s progress in Return@Work during those meetings and to support the employee if necessary [23].

#### Email Decision Aid for the Occupational Physician

Furthermore, the occupational physicians received automated email messages that were based on a decision aid with principles of stepped collaborative care. The decision aid supported the occupational physicians in the sickness guidance of the employees, in the monitoring of symptoms, functioning, and RTW. The outcomes of the monitor in Return@Work were used in the fully automated email messages for the occupational physician to give advice for stepped care treatment. Furthermore, the decision aid gave the occupational physician access to a consultant psychiatrist who, when needed, gave advice in case of stagnation [23].

#### Training

The occupational physicians in the intervention group were trained by the researchers and a consultant psychiatrist before recruitment of participants began. The training lasted half a day. In the training, occupational physicians were taught about the background and content of Return@Work and were instructed on how to guide employees through Return@Work and how to work with the decision aid. They were taught the basic principles of PST and CBT and how to apply these principles in the guidance of the employee.

#### Care as Usual

The occupational physicians in the control group provided usual sickness guidance to their employees. CAU was protocoled according to the guidelines of the NVAB [27]. However, several studies showed that adherence to this guideline was minimal [28,29]. For the process evaluation, actual provided care was assessed with a questionnaire by the participants in both groups.

### Outcomes

#### Overview

Data were collected by the research staff of The Netherlands Institute of Mental Health and Addiction. Participants completed online self-report questionnaires at baseline (T0) and at 3 (T1), 6 (T2), 9 (T3), and 12 months (T4) after inclusion. Data about RTW were derived from the registers of the occupational health service (Arbo Vitale) or employer (GGzBreburg).

#### Primary Outcome Measure

The primary outcome measure was duration until first RTW defined as the duration of sickness absence in calendar days from the day of randomization until the moment of first partial or full RTW. Subsequently, full RTW was analyzed. In accordance with the Dutch Sickness Benefits legislation, sickness absence within 4 weeks of full RTW was considered as belonging to the initial period of sickness absence. Furthermore, the total number of days of sickness absence in the first year follow-up period was tracked.

#### Secondary Outcome Measures

Secondary outcome measures were the severity of depression, anxiety, and somatization symptoms as measured with the PHQ-9, GAD-7, and PHQ-15 in terms of response and remission. Response was defined as a 50% reduction in symptoms on the PHQ-9, GAD-7, or PHQ-15, with the restriction that the baseline score on the questionnaire on which response was evaluated was above the cut-off point of 10 (otherwise it was defined as no response). Remission was defined as a score lower than 5 on the PHQ-9, GAD-7, or PHQ-15, with the restriction that the baseline score on the questionnaire on which remission was evaluated was above the cut-off point of 10 [24-26].

#### Covariates

All relevant covariates were measured at baseline. Demographic data such as age, gender, marital status, education level, and nationality were collected. Comorbid chronic medical illness was measured using a 28-item questionnaire developed by Statistics Netherlands. Job characteristics were measured by the Job Content Questionnaire (JCQ) [30]. Intention to RTW despite the existence of symptoms was measured on a 5-point Likert scale, with a response category varying from 1=certainly to 5=certainly not.

#### Process Outcomes

The actual health care utilization in both groups was assessed with the Trimbos/iMTA questionnaire for Costs associated with Psychiatric illness (TiC-P) [31]. The participants in the ECO condition received additional questions about the use of the intervention at the 3-month questionnaire. Furthermore, we recorded the number of log-ins per participant, the number of modules of the Return@Work intervention that they started, and the number of times the psychiatrist was consulted by the occupational physicians to assess adherence to the intervention.

### Data Analysis

All analyses were conducted according to the intention-to-treat principle. Baseline measurements of the participants were compared between the CAU and ECO condition using chi-square tests and independent samples *t* tests. The analyses of the primary outcomes, time to partial and full RTW, were performed with Kaplan-Meier time-to-event curves and Cox proportional hazards models. The shared-frailty procedure was used to account for clustering in the Cox proportional hazard models [32]. The Mann-Whitney *U* test was used to test the between-group difference in the average total number of sickness absence days during the 1-year follow-up.

Potential effect modification by severity of depression (PHQ-9), somatization (PHQ-15), and anxiety (GAD-7) at baseline as well as modification by company (Arbovitale and GGz Breburg) and intention to RTW in the presence of common mental disorder symptoms were evaluated. Interactions were tested for significance at the 5% significance level. Furthermore, a test of the proportional hazard assumption was conducted.

The analyses of the secondary outcomes were performed using multilevel logistic regression analysis with 3 levels: level of occupational physicians, level of employees within the cluster of occupational physicians, and level of number of measurements within the employees. First, the estimates of the intraclass correlation coefficients (ICCs) using the random intercept logistic-normal was assessed [33,34]. Then, the analysis of the outcomes was performed. For all analyses, all statistical tests were computed at the 5% significance level.

Per-protocol analyses were performed on the primary outcomes. In these analyses, the participants in the ECO condition who finished at least the introduction session of Return@Work were compared with the CAU participants.

The R package survival was used to test for clustering in the Cox regression analyses. The multilevel logistic regression analyses were performed in LME4 package of R [35]. All other analyses were performed in SPSS version 22.0 (IBM Corp, Armonk, NY, USA).

## Results

### Recruitment of Participants

**Figure 3:**
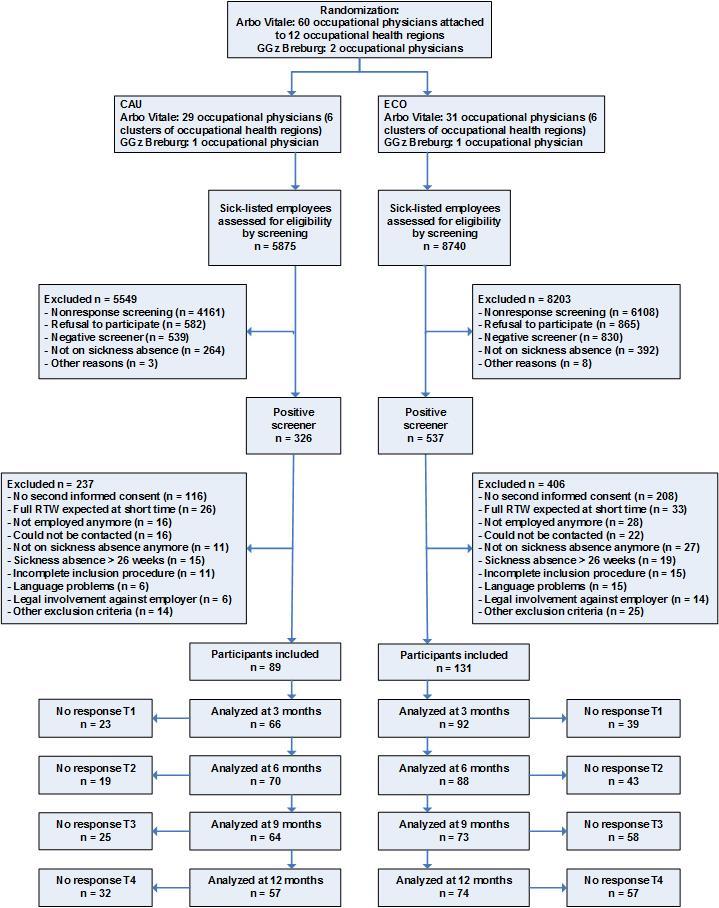
Flowchart of the clusters and participants.

In total, 14,615 all-cause sick-listed employees were approached between July 2011 and January 2013. Of this total group, 2232 of 14,615 employees (15.27%) participated in the screening. Of all 2232 screened participants, 863 (38.66%) positively screened for depression, somatization, or anxiety. Due to various reasons, 643 employees were excluded (see Figure 3). Finally, 220 employees who met all inclusion criteria were included in the study: 131 employees in the intervention condition and 89 employees in the control condition. The number of employees in the intervention and control condition were unequal due to the cluster randomization. Of all participants, 210 employees were included by the occupational health service (Arbo Vitale) and 10 employees by their employer (GGz Breburg). Figure 3 shows an overview of the recruitment flow.

### Loss to Follow-Up

Data about RTW were obtained from the registers of the OHS or employer. Sickness absence data were available for 86 employees in the control condition and for 130 employees in the intervention condition. For unknown reasons, the sickness absence data of 4 participants could not be found in the registers. These 4 participants did not differ significantly on average at baseline on sickness absence duration, depressive, somatization, or anxiety symptoms from the other participants.

For the self-reported secondary outcomes, follow-up questionnaires were returned by 158 of 220 participants (71.8%) at 3 months, 158 participants (71.8%) at 6 months, 137 participants (62.3%) at 9 months, and 131 participants (59.5%) at 12 months. At 9 months, the loss to follow-up rate was significantly higher in the ECO condition (44.3%, 58/131) than in the CAU condition (28%, 25/89, *P*=.02). However, the participants who did return the questionnaire at 9 months did not differ significantly at baseline on sickness absence duration, depression, somatization, or anxiety symptoms from the participants who did not return the questionnaire. This was the case in the ECO condition and in the control condition. From these results, we concluded that there was no evidence for selective dropout in this study.

### Baseline Characteristics

Table 1 shows a summary of the baseline characteristics of the participating employees. None of the baseline characteristics differed significantly between the intervention (ECO) and control (CAU) condition. This suggests that the randomization was successful.

As shown in Table 1, approximately half of the participants scored positive (≥10) on depression and somatization and anxiety symptoms (54%, 48/89 in the CAU group and 49.6%, 65/131 in the ECO group). Only 18.2% (40/220) of the participants scored positive on depressive, somatization, or anxiety symptoms alone (17%, 15/89 in the CAU group and 19.1%, 25/131 in the ECO group).

**Table 1:** Baseline characteristics of the participants in the care as usual (CAU) control and the ECO intervention groups (N=220).

Baseline characteristics			CAU (n=89)	ECO (n=131)	*P*
**Demographics**					
	Age (years), mean (SD)		45.5 (10.7)	43.4 (9.5)	.14
	Gender (female), n (%)		53 (60)	77 (58.8)	.91
	Married / living together, n (%)		62 (70)	91 (69.5)	.98
	**Educational level, n (%)**				.96
		Low	32 (36)	48 (36.6)	
		Average	31 (35)	47 (35.9)	
		High	26 (29)	36 (27.5)	
	Dutch nationality, n (%)		88 (99)	127 (96.9)	.65
**Symptoms and conditions**					
	**Common mental disorders symptoms, n (%)**				
		Only depressive symptoms (PHQ-9 ≥10)	5 (6)	11 (8.4)	.44
		Only somatization symptoms (PHQ-15 ≥10)	7 (8)	8 (6.1)	.61
		Only anxiety symptoms (GAD-7 ≥10)	3 (3)	6 (4.6)	.74
		Depressive and somatization symptoms	12 (14)	16 (12.2)	.78
		Depressive and anxiety symptoms	8 (9)	16 (12.2)	.45
		Somatization and anxiety symptoms	6 (7)	9 (6.9)	.97
		Depression, somatization and anxiety symptoms	48 (54)	65 (49.6)	.53
	Number of chronic medical conditions, mean (SD)		2.4 (3.0)	1.9 (1.7)	.10
**Job characteristics (JCQ), mean (SD)**					
	Decision latitude (range 24-96)		68.2 (10.6)	68.6 (12.3)	.81
	Psychological job demands (range 12-48)		33.4 (6.2)	34.6 (6.5)	.15
	Physical job demands (range 5-20)		10.9 (3.4)	11.6 (3.9)	.21
	Social support (range 8-32)		21.6 (4.2)	21.3 (4.0)	.61
	Job insecurity (range 3-12)		8.1 (0.8)	8.1 (0.9)	.97
**Sickness absence**					
	Duration at baseline in days, median (IQR)		70.0 (55.5-106.5)	73.0 (56.0-110.0)	.87
	Partial sickness absence at baseline, n (%)		27 (30)	36 (27.5)	.61
	Intention to RTW despite symptoms (range 1-5), mean (SD)		2.7 (1.3)	2.8 (1.2)	.53

### Primary Outcome

#### Overview

The shared-frailty procedure was used to account for clustering in the Cox proportional hazard models. The results, however, showed that there was no evidence of a clustering effect at the level of occupational physician regions (*P*=.92).

**Figure 4:**
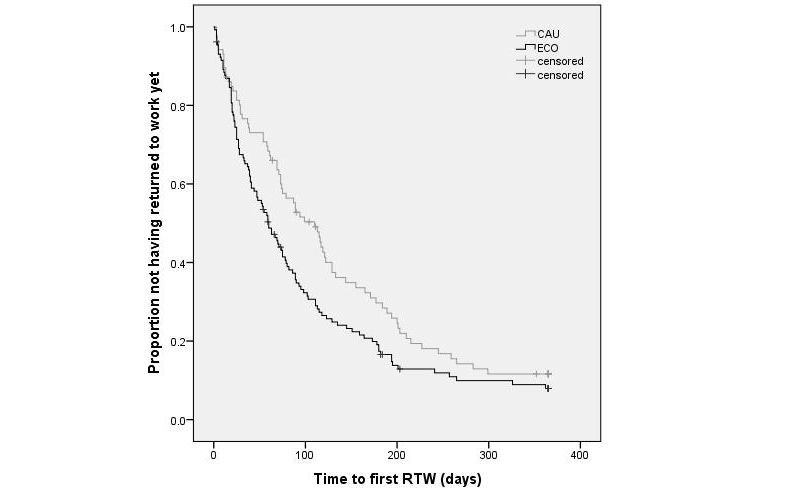
Kaplan-Meier curve of time to first partial or full return to work (RTW).

Figure 4 shows the Kaplan-Meier curves for the time until first RTW (partial or full) for both groups. Within the 1-year follow-up, 84% (72/86) of the CAU participants and 87.7% (114/130) of the ECO participants had achieved partial or full RTW. The median duration from baseline until first RTW (partial or full) was 77.0 days (IQR 29.0-152.3) in the CAU group and 50.0 days (IQR 20.8-99.0) in the ECO group (mean 99.0, SD 78.8 days and mean 72.5, SD 71.1 days, respectively). In total, 14 participants were censored because they resigned, 6 participants from the CAU group and 8 participants from the ECO group.

**Figure 5:**
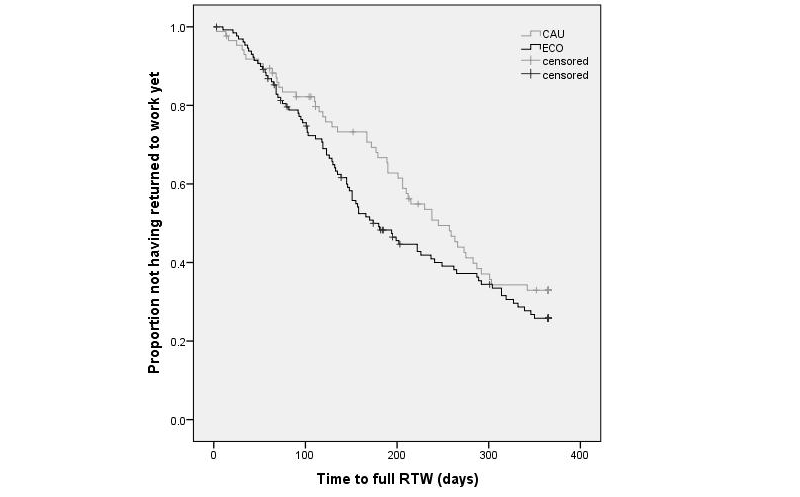
Kaplan-Meier curve of time to full return to work (RTW).

Figure 5 shows the Kaplan-Meier curves for the time to full RTW. In all, 61% (52/86) of CAU participants and 67.7% (88/130) of the ECO participants achieved full RTW within the 1-year follow-up. The median duration from baseline to full RTW was 178.0 days (IQR 72.0-243.3) in the CAU group and 131.0 days (IQR 68.5-198.0) in the ECO group (mean 164.8, SD 93.4 days and mean 146.3, SD 91.2 days, respectively).

The results of the Cox regression analysis on first RTW showed a significant effect of ECO intervention compared with usual care (HR 1.390, 95% CI 1.034-1.870, *P*=.03). The results of the Cox regression analysis on full RTW showed that the groups did not differ significantly from each other in duration until full lasting RTW (HR 1.287, 95% CI 0.913-1.814, *P*=.15). Because no differences were found between the CAU and ECO group for baseline characteristics, the Cox regression models were not adjusted for possible covariates.

To check whether the proportional hazard assumption was violated in the Cox regression analyses, log-minus-log plots were conducted. The log-minus-log plot for time to first RTW showed that the proportional hazard assumption was not violated. The log-minus-log curves of the CAU and ECO group for the time to full RTW crossed at approximately 40 days. Therefore, a Cox regression with time-dependent covariate was conducted. The time-dependent covariate was not significant (*P*=.26), indicating that the proportional hazard assumption for time to full RTW was also not violated.

The median total number of sickness absence days in the 1-year follow-up period was 228.0 days (IQR 111.0-365.0) in the CAU group and 174.0 days (IQR 100.0-321.0) in the ECO group (Mann-Whitney test; *P*=.10), and did not differ significantly between both groups (mean total number of sickness absence days was 225.3, SD 118.1 and mean 198.3, SD 116.0 days, respectively).

#### Effect Modification Primary Outcome

Having a depression (score ≥10 on the PHQ-9), somatization (score ≥10 on the PHQ-15), or anxiety disorder (score ≥10 on the GAD-7) at baseline were added separately as potential effect modifiers in the Cox proportional hazard model for first RTW and in the model for full RTW. No significant interaction effects were found.

Furthermore, the company (Arbo Vitale or GGz Breburg) and the intention to RTW despite having symptoms were added as potential effect modifiers in the Cox proportional hazard model for first RTW and in the model for full RTW. Again, no significant interaction effects were found.

### Secondary Outcomes

Table 2 shows the estimates of the ICCs using the random intercept logistic-normal model [33,34]. All ICCs were very close to zero. The largest ICC was found for response at 6 months, indicating that clusters explained 4.5% of the variance at most in the log odds-transformed outcome measures. Even though the ICC estimates suggested that the cluster effects were minor, we nevertheless used the random intercept logistic-normal model for estimating the effect of the treatment on the secondary outcomes to avoid inflated type I error rates.

**Table 2:** Estimated intraclass correlation coefficients (ICCs) for remission and response for each measurement occasion.^a^

Follow-up	Remission	Response
3 months	.007	.000
6 months	.000	.045
9 months	.000	.010
12 months	.038	.000

^a^ Intraclass correlations based on random intercept multilevel model [33].

Table 3 shows the percentage of employees in both groups who achieved remission and/or response. No significant differences between ECO and CAU were found for response. For remission, a significant difference was found at 9 months (T3) after baseline with the ECO group having a larger proportion achieving remission than the control group (OR 2.228, 95% CI 1.115-4.453, *P*=.02).

**Table 3:** Results on remission and response for both groups.

Follow-up	Remission				Response			
	CAU, n (%)	ECO, n (%)	OR^a^ (95% CI)	*P*	CAU, n (%)	ECO, n (%)	OR^a^ (95% CI)	*P*
3 months	16 (24)	25 (28)	1.180 (0.543-2.562)	.68	33 (50)	44 (49)	0.957 (0.507-1.806)	.89
6 months	20 (29)	36 (41)	1.731 (0.885-3.384)	.11	39 (56)	58 (66)	1.611 (0.694-3.742)	.27
9 months	23 (37)	41 (56)	2.228 (1.115-4.453)	.02	35 (56)	51 (70)	1.874 (0.879-3.996)	.12
12 months	25 (45)	36 (49)	1.157 (0.492-2.719)	.74	37 (66)	52 (70)	1.214 (0.576-2.556)	.61

^a^ Reference group is CAU.

### Process Outcomes

#### Health Care Utilization

Table 4 presents the proportion of participants in the CAU and ECO conditions that had contact with different health care professionals during the follow-up year. Generally, care in both groups consisted of contact with the occupational physician, general practitioner, and a mental health professional. There were no significant differences in health care use between the CAU and ECO participants.

**Table 4:** Health care utilization within 12 months after baseline.

Health care	CAU, n (%) (n=66)	ECO, n (%) (n=91)	*P*
Contact with occupational physician	61 (92)	81 (89)	.47
Contact with general practitioner	59 (89)	75 (82)	.22
Contact with mental health professional	51 (77)	63 (69)	.27
Day treatment for mental health problems	4 (6)	8 (9)	.53
Contact with social worker	6 (9)	6 (7)	.56
Participation in a self-help group	3 (5)	5 (6)	>.99

#### Adherence to the ECO Intervention

Of the 131 participants in the intervention group, 31 participants (23.7%) never logged in at Return@Work. Of the 100 participants who did log in at Return@Work, 10.0% (10/100) did not finish the introduction (which included information about Return@Work and a questionnaire). The mean number of total log-ins of the 90 participants who finished the introduction and actually started Return@Work was 7.8 (SD 6.1). Furthermore, 40% (36/90) of the participants minimally completed half of the modules of Return@Work.

For the 3-month questionnaire, 69 participants in the ECO condition answered additional questions about their experiences with Return@Work. Of these, 29% (20/69) reported that they discussed Return@Work with their occupational physician, initiated by themselves or their occupational physician. Furthermore, 15% (10/69) of the participants stated that Return@Work did not fit with their situation/problems, 61% (42/69) stated that Return@Work somewhat fit, and 24% (17/69) stated that Return@Work fit (quite) well. The psychiatrist was consulted only once by the occupational physicians.

### Per-Protocol Analyses

In the per-protocol analyses, the analyses on the primary outcomes were repeated, comparing the participants in the ECO condition who finished the introduction of Return@Work (n=90) with the CAU participants (n=89). The results of the per-protocol analyses did not differ from the results of the intention-to-treat analyses. The ECO participants who finished the introduction of Return@Work differed significantly from the CAU participants in duration until first RTW (HR 1.447, 95% CI 1.051-1.991; B=.369, SE=0.163; *P*=.02); however, they did not differ significantly from the CAU participants in duration until full RTW (HR 1.370, 95% CI 0.951-1.974; B=.315, SE=0.186; *P*=.09).

## Discussion

### Interpretation and Comparison With Other Studies

This study showed a positive effect of the ECO intervention on the duration until first RTW. On average, the participants in the ECO group returned to work (either partial or full) 27 days earlier than the participants in the control group receiving CAU did. Because eHealth focuses on the importance of RTW and on the employees’ perceptions regarding RTW with symptoms, we expected that the intervention would lead to a faster first RTW than CAU. However, we also expected that the partial RTW would lead to full RTW. On average, the participants in the ECO condition achieved full RTW 47 days earlier than the participants in the control condition, but this difference was not significant at the 5% level (2-tailed test). It may be that to reliably assess the effect on full RTW, larger comparison groups or a longer follow-up would be needed. However, another explanation may be that full RTW did not differ significantly between the 2 groups because the intervention primarily focused on enhancing partial RTW in a patient group that is known from the literature to have long-term full sickness absence and low full RTW. Time lag for RTW in patients with depression in remission has been found to be at least a year in general [36]. Maybe, in order to attain faster full RTW, the intervention should be longer and more explicitly focus on full RTW.

Additionally, Hees et al [37] examined the perspectives of some key stakeholders regarding the definition of successful RTW outcome after sickness absence due to common mental disorders. One of the results of this study was that the stakeholders did not necessarily consider full RTW as a prerequisite for successful RTW, but instead regarded a subjective criterion (ie, consensus between supervisor and employee) as more important for successful RTW [37]. Partial RTW could even be a long-term solution of employees with reduced work ability [38]. Because of the relatively long sickness duration of the participants at the start of our study, this could be the case in this study. Unfortunately, it is unknown whether partial RTW was a satisfactory outcome for all parties concerned.

The intervention was not intended to be a treatment for common mental disorders, but we expected that the feedback and support that the occupational physicians received from the decision aid would lead to a reduction of common mental disorder symptoms for the sick-listed employees. Also, a faster RTW might have a positive effect on the recovery from symptoms. For remission, at 9 months after baseline a significant difference was found between the 2 groups in favor of the ECO intervention. However, no effect was found on treatment response and the effect on remission did not persist through 12 months after baseline. This might be explained by the low adherence of the occupational physicians to the intervention. The eHealth module for the employee, Return@Work, was meant to be blended. The occupational physicians were instructed to inquire about the employee’s progress in Return@Work at the regular consults. However, the process outcomes showed that only 29% of the employees reported that they discussed the intervention with the occupational physician. Another signal of low adherence by the occupational physicians was the fact that only 1 occupational physician contacted the consultant psychiatrist during this study. It is unknown why the occupational physicians did not consult the psychiatrist more often. Another component of the ECO intervention was the decision aid. The decision aid supported the occupational physicians in the monitoring of symptoms, functioning, and RTW, and gave advice for stepped care treatment and referral to the curative sector. Unfortunately, it is unknown if the occupational physicians did adhere to the email messages from the decision aid. But the fact that the process outcomes showed that there were no differences in health care use between the ECO and CAU groups is an indication that the intervention did not lead to more referrals.

### Strengths and Limitations

This study discussed an innovative approach to reduce sickness absence because of its combination of an eHealth intervention aimed at RTW for sick-listed employees with a decision aid via email and the possibility of consulting a psychiatrist for the occupational physicians. However, the study design made it difficult to make inferences about the effectiveness of the different components of the intervention. However, there were several (mentioned previously) signals that the occupational physicians did not adhere to the intervention very well; therefore, it could be expected that the significant differences were mostly due to the Return@Work eHealth module.

The possible limited adherence of the occupational physician to the intervention could be caused by the design of this study. The participants were recruited by the researchers and the occupational physician was not informed (because of ethical reasons) about the participation of the employee until the employee started the eHealth module. The occupational physicians were informed by email and it is possible that they sometimes missed this notification. Furthermore, due to a reorganization by Arbo Vitale during this study, some of the sick-listed employees were not guided by 1 occupational physician, but by several occupational physicians. This was not helpful for the adherence of the occupational physicians to the ECO intervention.

Another limitation was that a relatively high proportion of the participants did not return 1 or more of the follow-up questionnaires. At 9 months, the loss to follow-up rate was significantly higher in the ECO condition than the CAU condition. Also at 9 months, significantly more participants in the ECO condition achieved remission than in the control condition. It might be the case that many participants who did not fill out the questionnaire at 9 months were not recovered, but it was also possible that the recovered participants did not feel the urge to fill out the questionnaires anymore. However, at baseline there were no differences between the participants who did or did not fill out the questionnaire at 9 months. Thus, no indications for selective dropout could be found in this study.

Furthermore, to achieve a successful RTW, it is important that all relevant stakeholders facilitate RTW [39]. A limitation of the ECO intervention was that the employers have no active role in the intervention. The cognitions that employees have about not being able to resume work while having symptoms is a cognition that employers/managers could also have. This could be one of the reasons why there was no effect of the ECO intervention on full RTW.

### Generalizability

A rather large population was screened for eligibility for participation in this study (N=14,615). From this population, 10,269 employees did not respond to screening, which might limit the generalizability of the findings of this study. It is unknown for what reasons employees did not respond. However, the employees who received a screener were on sickness absence for any cause and the focus of the study was explained as being on psychiatric symptoms, so it is possible that a large proportion of the nonresponders did not respond because they did not fit the description of the study. Also, it is possible that they may not have been on sickness absence anymore.

The participants in this study were mainly sick-listed employees of Dutch nationality, working in small- to medium-sized companies whose employer had insurance for the costs of sickness absence and sickness guidance. There is no indication that these employees would react differently to the intervention than employees from, for example, large companies. However, the organization of the sickness guidance in the company might have an effect on the ECO intervention. In this study, the sick-listed employees were not always guided by the same occupational physician. It might be the case that in larger companies where 1 occupational physician gives guidance to all sick-listed employees, the ECO intervention would be better guided by the sole occupational physician than the multiple occupational physicians did in this study. Continuity and accessibility of the occupational physician are important aspects for successful implementation of the ECO intervention.

### Conclusions

To our knowledge, this is the first study to combine an (eHealth) intervention specifically focused on RTW and cognitions regarding RTW while still having symptoms for sick-listed employees with common mental disorders with a decision aid for the occupational physician. It is promising that even though the adherence of the occupational physician to the ECO intervention was not optimal, ECO led to a faster first RTW and more remission of common mental disorder symptoms. This suggests that the potential of the ECO intervention might be better exploited with better continuity in and adherence of occupational physicians. Future research on optimizing the benefits of the ECO intervention should focus on improving the involvement of the occupational physician throughout the intervention, involving the employer/manager of the sick-listed employee, and monitoring the adherence of the occupational physicians to the decision aid.

## Multimedia Appendix 1

CONSORT-EHEALTH checklist V1.6.2 [40].

## Abbreviations

CAU: care as usual
CBT: cognitive behavioral therapy
ECO: E-health module embedded in Collaborative Occupational health care
HR: hazard ratio
ICC: intraclass correlation coefficient
JCQ: Job Content Questionnaire
PST: problem-solving treatment
RTW: return to work
RTW-SE: return-to-work self-efficacy
