# Supplementary material for: Effectiveness of a Blended Web-Based Intervention on Return to Work for Sick-Listed Employees With Common Mental Disorders: Results of a Cluster Randomized Controlled Trial
Source: J Med Internet Res. 2015 May 13;17(5):e116. doi: 10.2196/jmir.4097 (PMC4468600; doi:10.2196/jmir.4097)
Supplement: Supplementary file 1 [file jmir_v17i5e116_app1.pdf]

CONSORT-EHEALTH Checklist V1.6.2 Report

(based on CONSORT-EHEALTH V1.6), available at [http://tinyurl.com/consort-ehealth-v1-6].

4097

Date completed

12/1/2014 10:41:34

by

Danielle Volker

Effectiveness of a blended web-based intervention on return to work for sick-listed employees with common mental disorders (ECO): results of a cluster randomized controlled trial

TITLE

1a-i) Identify the mode of delivery in the title

"blended web-based intervention"

1a-ii) Non-web-based components or important co-interventions in title

There are no non web-based components or co-interventions

1a-iii) Primary condition or target group in the title

"for sick-listed employees with common mental disorders"

ABSTRACT

1b-i) Key features/functionalities/components of the intervention and comparator in the METHODS section of the ABSTRACT

"ECO is a guided web-based intervention with two parts: one for the employee, aimed at changing cognitions of the employee regarding RTW, and another part supporting the occupational physician (OP) with a fully-automated decision aid providing advice regarding treatment and referral options based on monitoring of the employees' progress during treatment."

1b-ii) Level of human involvement in the METHODS section of the ABSTRACT

1b-iii) Open vs. closed, web-based (self-assessment) vs. face-to-face assessments in the METHODS section of the ABSTRACT

1b-iv) RESULTS section in abstract must contain use data

"220 employees were included, 131 participants were randomized to the ECO-intervention and 89 to care as usual (CAU)."

1b-v) CONCLUSIONS/DISCUSSION in abstract for negative trials

This is not a negative trial

INTRODUCTION

2a-i) Problem and the type of system/solution

"Common Mental Disorders (CMDs), such as depressive, anxiety and somatization disorders, are strongly associated with long-term sickness absence."
"Besides the consequences of sickness absence for the individual employee, sickness absence also leads to substantial costs for society."
"Several studies have shown that a reduction of CMD symptoms was not enough to reduce sickness absence."
"Recent studies show the importance of factors like self-efficacy and the intention to resume work despite having symptoms"
"To our knowledge, no (e-health) intervention exists that specifically focuses on advancing RTW and cognitions regarding RTW for sick-listed employees with CMDs, combined with monitoring of progress in their mental health and a decision aid for the OP."

2a-ii) Scientific background, rationale: What is known about the (type of) system

"To our knowledge, no (e-health) intervention exists that specifically focuses on advancing RTW and cognitions regarding RTW for sick-listed employees with CMDs, combined with monitoring of progress in their mental health and a decision aid for the OP."

METHODS

3a) CONSORT: Description of trial design (such as parallel, factorial) including allocation ratio

"It is hypothesized that the ECO-intervention leads to a faster RTW and less CMD symptoms than usual care."

3b) CONSORT: Important changes to methods after trial commencement (such as eligibility criteria), with reasons

There were no important changes after start of the inclusion

3b-i) Bug fixes, Downtimes, Content Changes

There were no changes.

4a) CONSORT: Eligibility criteria for participants

"Employees (≥18 years of age) who were on sickness absence between 4 and 26 weeks and were screened positive (score ≥10) on either the depression scale of the Patient Health Questionnaire (PHQ-9), and/or the somatization scale of the Patient Health Questionnaire (PHQ-15) and/or the Generalized Anxiety Disorder questionnaire (GAD-7)"

4a-i) Computer / Internet literacy

4a-ii) Open vs. closed, web-based vs. face-to-face assessments:

"The participants were sick-listed employees in small-sized to medium-sized companies visiting their OP at Arbo Vitale (a large occupational health service) and sick-listed employees of GGz Breburg (a large mental health service employer) visiting their OP, both in The Netherlands."
"All employees on sickness absence due to any cause between 4 and 26 weeks who gave first informed consent were screened for depression (PHQ-9), somatization (PHQ-15) and anxiety (GAD-7). Employees who were considered as screen-positive on any of the three screening instruments were contacted by a research assistant, who was blinded for group assignment, by telephone. The research assistants checked for in- and exclusion criteria and gave information about the study."

4a-iii) Information giving during recruitment

4b) CONSORT: Settings and locations where the data were collected

"The participants were sick-listed employees in small-sized to medium-sized companies visiting their OP at Arbo Vitale (a large occupational health service) and sick-listed employees of GGz Breburg (a large mental health service employer) visiting their OP, both in The Netherlands."

4b-i) Report if outcomes were (self-)assessed through online questionnaires

"Participants completed online self-report questionnaires at baseline (T0) and at three (T1), six (T2), nine (T3) and twelve months (T4) after inclusion."

4b-ii) Report how institutional affiliations are displayed

5) CONSORT: Describe the interventions for each group with sufficient details to allow replication, including how and when they were actually administered

5-i) Mention names, credential, affiliations of the developers, sponsors, and owners

5-ii) Describe the history/development process

5-iii) Revisions and updating

There was no revision

5-iv) Quality assurance methods

5-v) Ensure replicability by publishing the source code, and/or providing screenshots/screen-capture video, and/or providing flowcharts of the algorithms used

5-vi) Digital preservation

5-vii) Access

"The employee received an individual login code for the E-health RTW module Return@Work. "

5-viii) Mode of delivery, features/functionalities/components of the intervention and comparator, and the theoretical framework

"Return@Work included the following five modules: psycho-education, a module aimed at cognitions with regard to RTW while having symptoms (based on cognitive-behavioural principles, CBT), a module aimed at increasing problem solving skills with Problem Solving Treatment (PST) exercises, a module for pain and fatigue management and for reactivation and a module for relapse prevention."

"the OPs received automated email messages that were based on a decision aid with principles of stepped, collaborative care."

5-ix) Describe use parameters

5-x) Clarify the level of human involvement

"The employees worked through Return@Work individually, but were free to discuss topics or assignments with the OP. OPs were asked to follow the guidelines of the Dutch Board for Occupational Medicine (NVAB), thus, as in usual sickness guidance, the OP and employee met each other face to face on a regular basis."

5-xi) Report any prompts/reminders used

There were no prompts/reminders

5-xii) Describe any co-interventions (incl. training/support)

"OPs in the intervention group were trained by the researchers and a consultant psychiatrist prior to the start of the recruitment of participants....etc"

6a) CONSORT: Completely defined pre-specified primary and secondary outcome measures, including how and when they were assessed

"Data about RTW were derived from the registers of the occupational health service (Arbo Vitale) or employer (GGzBreburg)."

"The primary outcome measure was duration until first RTW; defined as the duration of sickness absence in calendar days, from the day of randomization until the moment of first partial or full RTW. Subsequently, full RTW was analyzed."

"Secondary outcome measures were the severity of depressive, anxiety and somatization symptoms, as measured with the PHQ-9, GAD-7 and PHQ-15 in terms of response and remission... etc"

6a-i) Online questionnaires: describe if they were validated for online use and apply CHERRIES items to describe how the questionnaires were designed/deployed

6a-ii) Describe whether and how “use” (including intensity of use/dosage) was defined/measured/monitored

6a-iii) Describe whether, how, and when qualitative feedback from participants was obtained

6b) CONSORT: Any changes to trial outcomes after the trial commenced, with reasons

There were no changes

7a) CONSORT: How sample size was determined

7a-i) Describe whether and how expected attrition was taken into account when calculating the sample size

7b) CONSORT: When applicable, explanation of any interim analyses and stopping guidelines

"Per-protocol analyses were performed on the primary outcomes. In these analyses the participants in the ECO condition who finished at least the introduction session of Return@Work were compared with the CAU participants."

8a) CONSORT: Method used to generate the random allocation sequence

"The clusters of OPs were randomized by an independent statistician using a computer algorithm for randomization. Six regions (31 OPs) were allocated to the ECO group and six regions (29 OPs) were allocated to the control group."

8b) CONSORT: Type of randomisation; details of any restriction (such as blocking and block size)

"The clusters of OPs were randomized by an independent statistician using a computer algorithm for randomization. Six regions (31 OPs) were allocated to the ECO group and six regions (29 OPs) were allocated to the control group."

9) CONSORT: Mechanism used to implement the random allocation sequence (such as sequentially numbered containers), describing any steps taken to conceal the sequence until interventions were assigned

"The clusters of OPs were randomized by an independent statistician using a computer algorithm for randomization. Six regions (31 OPs) were allocated to the ECO group and six regions (29 OPs) were allocated to the control group."

10) CONSORT: Who generated the random allocation sequence, who enrolled participants, and who assigned participants to interventions

"The clusters of OPs were randomized by an independent statistician using a computer algorithm for randomization. Six regions (31 OPs) were allocated to the ECO group and six regions (29 OPs) were allocated to the control group."

"The research assistants and the participants were blind for the allocation when assessing the eligibility of sick-listed employees for participating in this study."

"the participant was informed by the researchers per telephone about the allocation."

11a) CONSORT: Blinding - If done, who was blinded after assignment to interventions (for example, participants, care providers, those assessing outcomes) and how

11a-i) Specify who was blinded, and who wasn’t

"Because the OPs had to guide the intervention, they could not be blinded for the group assignment after randomization. However, they participated in only one experimental condition: either ECO, or CAU. The research assistants and the participants were blind for the allocation when assessing the eligibility of sick-listed employees for participating in this study."

11a-ii) Discuss e.g., whether participants knew which intervention was the “intervention of interest” and which one was the “comparator”

11b) CONSORT: If relevant, description of the similarity of interventions

not relevant

12a) CONSORT: Statistical methods used to compare groups for primary and secondary outcomes

"The analyses of the primary outcomes, time to partial and full RTW, were performed with Kaplan-Meier time-to-event curves and Cox proportional hazards models. The shared-frailty procedure was used to account for clustering in the Cox proportional hazard models"
"The analyses of the secondary outcomes were performed using multilevel logistic regression analysis with 3 levels; level of OPs, level of employees within the cluster of OPs and level of number of measurements within the employees."

12a-i) Imputation techniques to deal with attrition / missing values

we did not impute missing data

12b) CONSORT: Methods for additional analyses, such as subgroup analyses and adjusted analyses

"Per-protocol analyses were performed on the primary outcomes. In these analyses the participants in the ECO condition who finished at least the introduction session of Return@Work were compared with the CAU participants."

RESULTS

13a) CONSORT: For each group, the numbers of participants who were randomly assigned, received intended treatment, and were analysed for the primary outcome

See figure1 flowchart of participants

13b) CONSORT: For each group, losses and exclusions after randomisation, together with reasons

See figure1 flowchart of participants

13b-i) Attrition diagram

14a) CONSORT: Dates defining the periods of recruitment and follow-up

"In total, 14,615 all cause sick-listed employees were approached between July 2011 and January 2013"

14a-i) Indicate if critical “secular events” fell into the study period

14b) CONSORT: Why the trial ended or was stopped (early)

the trial was ended or stopped

15) CONSORT: A table showing baseline demographic and clinical characteristics for each group

See table 1 with baseline characteristics

15-i) Report demographics associated with digital divide issues

See table 1 with baseline characteristics

16a) CONSORT: For each group, number of participants (denominator) included in each analysis and whether the analysis was by original assigned groups

16-i) Report multiple “denominators” and provide definitions

This is reported. See result section

16-ii) Primary analysis should be intent-to-treat

"All analyses were conducted according to the intention-to-treat principle"

17a) CONSORT: For each primary and secondary outcome, results for each group, and the estimated effect size and its precision (such as 95% confidence interval)

This is reported. See result section

17a-i) Presentation of process outcomes such as metrics of use and intensity of use

See subhead "Adherence to the ECO intervention" in the result section

17b) CONSORT: For binary outcomes, presentation of both absolute and relative effect sizes is recommended

Hazard Ratio's and Odds Ratio's are reported

18) CONSORT: Results of any other analyses performed, including subgroup analyses and adjusted analyses, distinguishing pre-specified from exploratory

"In the per-protocol analyses, the analyses on the primary outcomes were repeated, comparing the participants in the ECO condition who finished the introduction of Return@Work (n=90) with the CAU participants (n=89)."

18-i) Subgroup analysis of comparing only users

"In the per-protocol analyses, the analyses on the primary outcomes were repeated, comparing the participants in the ECO condition who finished the introduction of Return@Work (n=90) with the CAU participants (n=89)."

19) CONSORT: All important harms or unintended effects in each group

There were no harms or unintended effects.

19-i) Include privacy breaches, technical problems

19-ii) Include qualitative feedback from participants or observations from staff/researchers

DISCUSSION

20) CONSORT: Trial limitations, addressing sources of potential bias, imprecision, multiplicity of analyses

20-i) Typical limitations in ehealth trials

See subhead "Strengths and limitations"

21) CONSORT: Generalisability (external validity, applicability) of the trial findings

21-i) Generalizability to other populations

See subhead "generalizability"

21-ii) Discuss if there were elements in the RCT that would be different in a routine application setting

"The participants were recruited by the researchers and the OP was not informed (because of ethical reasons) about the participation of the employee until the employee started the e-health module. The OPs were informed by email and it is possible that they sometimes missed this notification."

22) CONSORT: Interpretation consistent with results, balancing benefits and harms, and considering other relevant evidence

22-i) Restate study questions and summarize the answers suggested by the data, starting with primary outcomes and process outcomes (use)

"The present study shows a positive effect of the ECO intervention on the duration until first RTW. On average, the participants in the ECO group returned to work (either partial or full) 27 days earlier than the participants in the control group receiving CAU. Because the e-health focuses on the importance of RTW and on the employees’ perceptions regarding RTW with symptoms, we expected that the intervention would lead to a faster first RTW than CAU... etc"

22-ii) Highlight unanswered new questions, suggest future research

Other information

23) CONSORT: Registration number and name of trial registry

" Netherlands Trial Register NTR2108."

24) CONSORT: Where the full trial protocol can be accessed, if available

"The design of this study has been extensively described in Volker et al"

Volker D, Vlasveld MC, Anema JR, Beekman ATF, Hakkaart-van Roijen L, Brouwers EPM et al. Blended E-health module on return to work embedded in collaborative occupational health care for common mental disorders: design of a cluster randomized controlled trial. Neuropsychiatr Dis Treat 2013; 9:529-537

25) CONSORT: Sources of funding and other support (such as supply of drugs), role of funders

"This study is funded by The Netherlands Organization for Health Research and Development (ZonMw) and by Achmea, a Dutch insurance company. The results and conclusions reported in this paper are independent from the funding sources."

X26-i) Comment on ethics committee approval

x26-ii) Outline informed consent procedures

X26-iii) Safety and security procedures

X27-i) State the relation of the study team towards the system being evaluated
